# A comparative study of the nutritional and physiological potential of Xuta (edible *Jatropha curcas* L.) protein: Insights into its digestibility and effects on the intestinal barrier

**DOI:** 10.1016/j.crfs.2025.101257

**Published:** 2025-12-01

**Authors:** Mona Grünwald, Nabil Adrar, George Francis, Nils Rugen, Matthias Döring, Hans-Peter Braun, Tuba Esatbeyoglu

**Affiliations:** a Department of Molecular Food Chemistry and Food Development, Institute of Food and One Health, Gottfried Wilhelm Leibniz University Hannover, Am Kleinen Felde 30, Hannover, 30167, Germany; b Jatropower AG, Haldenstrasse 5, Baar, CH-6340, Switzerland; c Department of Plant Proteomics, Institute of Plant Genetics, Gottfried Wilhelm Leibniz University Hannover, Herrenhäuser Straße 2, Hannover, 30419, Germany

**Keywords:** Alternative protein, Sustainability, Digestibility, Proteomics, Nutritional quality, Food safety

## Abstract

The growing demand for alternative plant-based proteins, coupled with the limitations of the sources currently available, highlights the importance of exploring novel options such as Xuta (edible *Jatropha curcas* L.), a promising though little known protein-rich crop. In this study, the quality, digestibility, and amino acid (AA) profiles of Xuta proteins were investigated, focusing on the influence of cultivar and harvest year, and comparing the results with those of common plant-based protein sources. Protein digestibility was assessed using the standardised *in vitro* digestion model INFOGEST, with the protein profiles analysed at each stage of digestion. Shotgun proteomics was performed to compare the protein composition across the digestive phases. Post-digestion AA profiles and their transepithelial transport were determined using a Caco-2/HT29-MTX intestinal co-culture model. The effects on the intestinal barrier integrity were assessed via transepithelial electrical resistance and paracellular permeability measurements. Xuta proteins were found to be comparable in digestibility to conventional plant proteins, resulting in high peptide and AA release during digestion and good essential AA profiles, while digestibility varied by harvest year. Notably, Xuta digests had no adverse effects on intestinal barrier function. These findings suggest that Xuta is a valuable alternative protein source offering favourable digestibility without compromising intestinal barrier integrity.

## Highlights

• The protein quality of Xuta varied primarily with the harvest year rather than the variety.
• *In vitro* digestion showed effective protein hydrolysis and good amino acid bioaccessibility.
• Digestion products enhanced epithelial barrier integrity *in vitro*.
• Methionine was the limiting essential amino acid, while arginine is particularly abundant in Xuta.
• Combining Xuta with methionine-rich plant proteins can improve its amino acid profile.

## Graphical abstract

**Figure:**
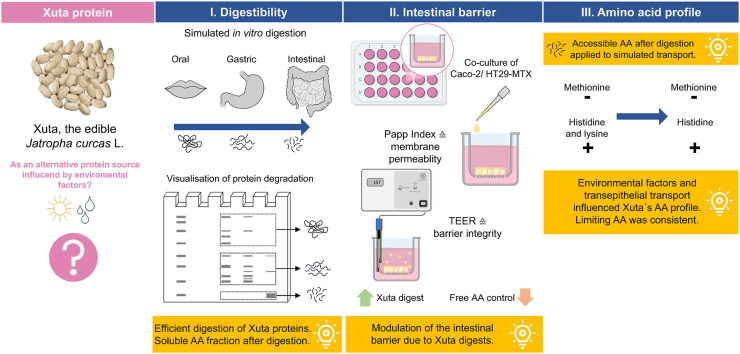

## 1 Introduction

The combined pressures of global population growth and climate change are intensifying the search for sustainable and resilient food systems. Within this context, alternative protein sources, particularly plant-based proteins, are attracting growing scientific and commercial interest (Ferrara et al., 2025; Malek and Umberger, 2023). As environmentally friendly and nutritionally promising substitutes for animal proteins plant-based foods generally exhibit lower greenhouse gas emissions and reduced resource demands compared to animal production systems (Fasolin et al., 2019). However, many existing plant-based options face challenges regarding protein digestibility and require thorough safety assessments as they often contain antinutritional factors, particularly when considering the development and use of novel protein sources. Although specific processing strategies can improve digestibility or mitigate potential risks, comprehensive evaluations remain scarce (Milana et al., 2025).

The kernels of the edible *Jatropha curcas* L., known as Xuta, a member of the Euphorbiaceae family, are primarily cultivated in the Totonacapan region of Mexico (Yucatan, northern Puebla, and Veracruz) but have also been introduced to parts of Africa and Asia. Xuta has recently gained attention as a novel protein source following their approval by the European Food Safety Authority (EFSA et al., 2022). Traditionally consumed in Mexican cuisine, Xuta kernels are characterised by their high protein content and therefore justify an in-depth study of their nutritional and functional properties (Martínez-Herrera et al., 2006). In plantations under suboptimal soil and climatic conditions in India, mature stands yielded of over 2000 kg/ha/year (Francis et al., 2013, 2019; Devappa et al., 2010). The species is well adapted to harsh environments, tolerating a wide temperature range (20–38 °C) and low annual rainfall below 800 mm. Although the Indian region in which the analysed seeds were cultivated benefits from two rainy seasons (June–August and October–December), total annual rainfall from 2022 to 2024 remained below 500 mm. Under such conditions, it is recommended that plants be given an additional 15 L of water every 15 days during rain-free months (Francis et al., 2013). Xuta kernels contain more than 50 % fat, depending on the cultivar, and their defatted meal retains over 55 % protein (Martínez-Herrera et al., 2006; Francis et al., 2013). The crude protein content of native kernels varies substantially by region (18.8–33.3 %), and even greater variability is reported among more than 70 cultivars grown worldwide (Martínez-Herrera et al., 2010; Basha et al., 2009). A comprehensive amino acid (AA) profile analysis of Mexican-grown Xuta revealed glutamic acid as the most abundant, followed by aspartic acid and arginine. Among the essential amino acids (EAAs), leucine was the most abundant, whereas methionine was the least abundant. The high leucine content is nutritionally relevant, as branched-chain AA (BCAAs), particularly leucine, play a key role in stimulating muscle protein synthesis and metabolic signaling (Devries et al., 2018). Agroclimatic conditions appear to have a minimal influence on the AA composition (Martínez-Herrera et al., 2006).

To date, studies on Xuta proteins have focused exclusively on native kernel material, without evaluating protein quality using standardized *in vitro* digestion protocols such as the INFOGEST method (Brodkorb et al., 2019), which are essential for assessing protein digestibility and post-digestive nutritional properties. During simulated digestion, proteins remain structurally intact during the oral phase, undergo pepsin-mediated hydrolysis in the gastric phase, and are further degraded by pancreatin (primarily trypsin) in the intestinal phase (Brodkorb et al., 2019; Goksen et al., 2025; Grundy et al., 2025). The extent of hydrolysis depends on the physicochemical and colloidal properties of the protein matrix. For instance, casein micelles can coagulate under acidic conditions, forming clots that slow pepsinolysis and delay AA release, while denatured whey proteins are more rapidly degraded (Loveday, 2019; Ye et al., 2016). Protein digestibility is shaped by both intrinsic factors (AA composition, folding, aggregation) and extrinsic factors (pH, temperature, ionic strength, and interactions with antinutrients). Moderate thermal treatment enhances digestibility by unfolding proteins and inactivating inhibitors, whereas excessive heat can cause aggregation and reduce enzyme accessibility. These matrix-related effects ultimately determine AA bioaccessibility and bioavailability, highlighting the need for standardized digestion protocols to ensure comparability across studies (Auer et al., 2024; Orlien et al., 2023; Sá et al., 2020). In the intestinal phase, proteolysis is highly efficient and generates mainly di- and tripeptides, although some resistant or bioactive peptides may persist and influence epithelial function (Loveday, 2019).

Protein digestibility and AA availability are key determinants of nutritional quality (Ferrara et al., 2025; Sousa et al., 2023). In this context, digestibility refers to the enzymatic hydrolysis and luminal disappearance of protein and peptide structures during gastrointestinal transit. Bioaccessibility describes the fraction of peptides and AAs that become solubilized and accessible to digestive enzymes in the gastrointestinal lumen, thus representing the pool potentially available for absorption. Bioavailability, in contrast, refers to the fraction that is absorbed through the intestinal epithelium and becomes available for metabolic utilization in target tissues (Goksen et al., 2025; Dima et al., 2020; Grundy et al., 2024). While animal proteins such as casein, whey, and egg typically exhibit high digestibility and complete EAA profiles, often reflected in the digestible indispensable amino acid score (DIAAS) of over 100 %, plant proteins show greater variability in both digestibility and DIAAS (Broucke et al., 2025; Singh et al., 2025). Therefore, comparative metrics such as DIAAS, EAA content, and identification of the limiting amino acid (LAA) are critical for evaluating novel protein sources (Ariëns et al., 2021).

Although INFOGEST is widely applied to assess bioaccessibility, standardised approaches for studying intestinal absorption are lacking. Bioaccessibility studies are often combined with *in vitro* intestinal epithelial co-culture models (e.g., Caco-2/HT29-MTX) to simulate human intestinal physiology and provide insights into bioavailability (Arranz et al., 2017; Hoffmann et al., 2021; Araújo and Sarmento, 2013). Barrier integrity is commonly monitored through transepithelial electrical resistance (TEER) and paracellular permeability measurements, which reflect tight junction integrity and overall epithelial function (Srinivasan et al., 2015; Shaughnessey et al., 2022; Gonschior et al., 2022; Bonnans et al., 2014). Together, these methods enable the mechanistic evaluation of nutrient digestion, absorption, and their effects on epithelial integrity - factors that are essential for nutritional assessment and safety of novel foods such as Xuta kernels (Kondrashina et al., 2024).

This study provides the first comprehensive evaluation of Xuta protein digestibility using the standardised INFOGEST protocol, with comparative analysis of the resulting AA profiles against established plant-based proteins. It is also the first study to investigate Xuta protein digests in an intestinal co-culture model, assessing both AA transport and potential effects on intestinal barrier integrity. Additionally, the influence of cultivar and harvest year on Xuta protein composition is examined, addressing significant knowledge gaps concerning its nutritional characterisation and safe use as a novel food ingredient.

## 2 Materials and methods

### 2.1 Samples

Three Xuta varieties (JPNT1, JPNT2, and JPNT3) harvested in three consecutive years (2022, 2023, and 2024) were analysed. The harvested seeds were sun-dried for 2–3 days, de-hulled immediately, and the kernels dried further on plastic or fibre mats under direct sunlight until the moisture content was below 6 %. The kernels were then stored in jute sacks out of direct sunlight. All seeds were sourced from *Jatropower*'s research farms in India (see Table 1 for details). While two harvest periods occur annually (July–September and November–February), all samples used in this study were obtained from the July–September harvests.

**Table 1:** Origin of Xuta cultivars used in this study.

Cultivar name	Crop farms	Age of the plants in 2022
JPNT1	A.S. Kulam Village, Coimbatore district, Tamil Nadu, India	1 year
JPNT2	A.S. Kulam Village, Coimbatore district, Tamil Nadu, India	1 year
JPNT3	Illuppunagaram Village, Tiruppur District, Tamil Nadu, India	4 years

To illustrate the cultivation conditions, data for JPNT3 is provided: Annual rainfall at the plantation site was 345 mm in 2022, 155 mm in 2023, and 487 mm in 2024. During rain-free months, irrigation was applied at 15 L per plant every 15 days. The soil, classified as sandy loam, is nutrient-poor (nitrogen 58.8 kg/acre, phosphate 3.5 kg/acre, potassium 500 kg/acre), with an electrical conductivity of 0.14 dSm^−1^, pH 7.8, and organic matter content 0.8 %. To compensate for these nutrient deficiencies, each plant received 40 g urea, 120 g superphosphate, and 30 g muriate of potash annually.

To provide a point of comparison, the following established plant-based protein sources were included: almonds (*Prunus dulcis*) and cashews (*Anacardium occidentale*) as representatives of nuts; soybeans (*Glycine max*), green peas (*Pisum sativum*), red lentils (*Lens culinaris*), and lupins (*Lupinus albus*) as representatives of legumes; and oats (*Avena sativa*) as a cereal representative, which are widely used in the production of meat and dairy alternatives. All comparative samples were purchased in dried form from commercial retailers in Germany.

### 2.2 Chemicals

All chemicals used were of analytical grade and procured from German suppliers. Sulfuric acid (nitrogen-free, Kjeldahl grade), calcium chloride dihydrate (CaCl_2_(H2O)_2_), hydrochloric acid (HCl), sodium hydroxide (NaOH), human salivary amylase, porcine pancreatin (4x USP), bile salts, trichloroacetic acid, di-sodium tetraborate decahydrate, sodium dodecyl sulfate (SDS), *ortho*-phthalaldehyde (OPA), dithiothreitol (DTT), L-serine, Pierce bicinchoninic acid (BCA) protein assay kit, 2-mercaptoethanol, glycine, bromthymol blue, Tris, acrylamide, ammonium persulfate, tetramethylethylenediamine, protein ladder (BLUeye Prestained Protein), ROTI®Blue (for preparing Coomassie brilliant blue), sodium fluorescein, phosphate buffer saline (PBS), potassium carbonate, sodium acetate buffer, methanol (HPLC grade), acetic acid (100 %), acetonitrile (LC-MS grade), formic acid (LC-MS grade) and amino acid analytical standard (dissolved in 0.1 M HCl).

The rabbit gastric extract was provided by Lipolytech (Marseille, France), the protease inhibitor 4-(2-aminoethyl) benzensulfonylfluoride (AEBSF, trademark Pefabloc®) was obtained from Roche (Basel, Switzerland), and 3-mercaptopropionic acid (99 %) was purchased from Alfa Aesar (Haverhill, Massachusetts, USA).

For the cell culture studies, Dulbecco's modified Eagle's medium (DMEM) with 4.5 g/L glucose, 0.87 g/L stable glutamine, 0.11 g/L sodium pyruvate, and 3.7 g/L NaHCO_3_, 10 % (v/v) fetal bovine serum, 1 % (v/v) penicillin/streptomycin and 1 % (v/v) non-essential amino acids (100x), trypsin/EDTA were all purchased from Pan-Biotech, Aidenbach, Germany and Triton X-100™ was obtained from Merck (Darmstadt, Germany). Double deionized water (ultra-pure water) was obtained using PURELAB® flex 3 (ELGA LabWater, Veolia Water Technologies Deutschland, Celle, Germany).

### 2.3 Determination of crude protein content

The crude protein content was determined by measuring the nitrogen content according to the Kjeldahl method following DIN EN ISO 20483. The samples were digested with 98 % sulfuric acid at 410 °C, followed by steam distillation. The released ammonia was captured in a 2 % boric acid solution and subsequently titrated with 0.05 M sulfuric acid. The crude protein content was calculated as a percentage using a conversion factor of 5.8 (Equation (1)) (Pirgozliev et al., 2012):

(1) $$\text{Crude}\;\text{protein}\;\left(\%\right)=\left(\frac{V_{\text{Titrated}\;H_{2}SO_{4}}\times M_{H_{2}SO_{4}}\times 1.4007}{m_{\text{sample}}}\right)\times c\text{onversion}\;\text{factor}$$

### 2.4 Simulated *in vitro* digestion

According to Brodkorb et al. (2019), the INFOGEST method was used to mimic the physiological conditions in the human stomach and intestine for *in vitro* digestion. All enzymes were tested for their activity prior to the experiment.

A hydrothermal treatment recommended by the EFSA (European Food Safety Authority, 2022) for safe human consumption was applied prior to the digestion by treating the kernels for 40 min at 100 % humidity at 125 °C in a combi steamer (iCombi Classic-LM200B, Rational, Landsberg am Lech, Germany). After treatment, the kernels were left to air-dry at room temperature for 10 min and subsequently cooled at 4 °C for another 10 min. All kernels (untreated and treated) were ground in a blender (BlendTec Classic 575; LUBA, Bad Homburg v.d.H, Germany) and stored at 4 °C.

A sample amount corresponding to 0.2 g protein (prior determined by the Kjeldahl method) is weighed, based on a total volume of 40 mL (Sousa et al., 2020). For the oral phase, the sample was mixed with 4 mL ultra-pure water using an Ultra-Turrax® at a maximum rotation speed for 30 s. The resulting paste-like sample was then diluted with 4 mL simulated salivary fluid (SSF) containing 1.5 mM CaCl_2_(H2O)_2_. Under constant agitation, the pH was adjusted to 7.0 with 1 M NaOH using an automatic titration device (Titroline 7750, Xylem Analytics, Weilheim, Germany). The appropriate amount of human salivary amylase was then added to achieve an activity of 75 U/mL. To accomplish a 1x concentration of SSF, the mixture was filled up to 10 mL with water and was incubated for 2 min at 37 °C. A sample of 2 mL each was collected. Afterwards, 6.4 mL of pre-warmed simulated gastric fluid (SGF) was added, which contained a lipase activity of 60 U/mL and a pepsin activity of 2000 U/mL due to the rabbit gastric extract used, and 0.15 mM CaCl_2_(H_2_O)_2_ in the final digestion mixture. The pH value was adjusted to 3.0 using 1 M HCl solution, and the mixtures were filled up to 16 mL with ultra-pure water. The samples were again incubated at 37 °C for 2 h with sufficient shaking. At this point, 2 mL samples were withdrawn. For the final intestinal phase, simulated intestinal fluid (SIF) was added to the mixture in a ratio of 1:1 (v/v), which corresponds to a volume of 7.7 mL. After adjusting the pH to 7.0 using 1 M NaOH solution, a final concentration of 10 mM bile salts was added. The samples were then incubated for 30 min at 37 °C with constant stirring. Subsequently, CaCl_2_(H_2_O)_2_ was then added to reach a concentration of 0.6 mM in SIF, followed by the corresponding volume of porcine pancreatin to give a final mixture with an activity of 100 U/mL trypsin. The pH was then adjusted to 7.0 using 1 M NaOH, after which the mixture was filled up to a final volume of 28 mL, and incubated for 2 h at 37 °C with constant shaking. Digestion was stopped by adding the 4-(2-aminoethyl) benzensulfonylfluoride (AEBSF) protease inhibitor, reaching a final concentration of 0.05 mM after 120 min of the intestinal phase. All the samples were then immediately snap frozen in liquid nitrogen. After defrosting, the samples were separated into soluble (S) and insoluble (P) fractions by centrifugation at 4500 rpm and 4 °C for at least 30 min, with the supernatant and pellet being collected, respectively. The S samples were analysed further.

### 2.5 Determination of free amino acid content

According to Nielsen et al. (2001) and Jadhav et al. (2021) the OPA method was used with modifications to evaluate the amount of free amino groups, which reflect the degree of protein hydrolysis. The freeze-dried, separately digested oral, gastric, and intestinal phase samples were weighed into a reaction tube and diluted with ultra-pure water to a concentration of 10 mg/mL. The sample was then mixed with 5 M trichloroacetic acid and ultra-pure water at a ratio of 1:1:3 (*v/v/v*) in a reaction tube, incubated at 4 °C for 15 min, and centrifuged at 18.000 rpm for 5 min at RT. The OPA reagent was prepared using 1.90 g of di-sodium tetraborate decahydrate, 50 mg SDS, 40 mg OPA (previously dissolved in 1 mL pure ethanol), and 44 mg DTT dissolved in 50 mL ultra-pure water. A dilution series of serine (0.25–10 μg/mL) was prepared in ultra-pure water. Twenty-five microliters of sample were pipetted in a 96-well plate and 175 μL of OPA reagent was added. The 96-well microplate was then incubated in the dark for 2 min, and the fluorescence intensity was measured immediately using a microplate reader (Infinite® M200, Tecan, Männedorf, Switzerland) with an excitation wavelength of 350 nm and an emission wavelength of 450 nm. The results were expressed as serine equivalents and calculated using a serine calibration curve (10–0.25 μg/mL) with linear regression (R^2^ = 0.996; LOD = 0.36 μg/mL; LOQ = 1.08 μg/mL).

### 2.6 Electrophoretic profiling of digested Xuta protein (SDS-PAGE)

The freeze-dried, separately collected oral, gastric, and intestinal phase digested sample supernatants were weighed into a reaction tube and diluted with ultra-pure water to a concentration of 10 mg/mL. The content of soluble proteins in the solution was determined by bicinchoninic acid (BCA) using the Pierce BCA Protein Assay Kit according to the manufacturer's instructions. A solution containing a volume equivalent to a total of 10 μg of protein was mixed at a ratio of 1:1 (*v/v*) with Laemmli buffer (4 % SDS, 10 % 2-mercaptoethanol, 20 % glycine, 0.004 % bromthymol blue, and 125 mM Tris) in a reaction tube. This mixture was then heated for 5 min at 95 °C using a thermoshaker (Cellmedia, Zeitz, Germany). After cooling to RT for 10 min, the samples were loaded onto a polyacrylamide gel. The gel consists of a resolving gel (40 % acrylamide, resolving buffer (1.5 M tris, pH 8.8), ultra-pure water, 0.8 % ammonium persulfate, and 0.08 % tetramethylethylenediamine (TEMED)) and a stacking gel (15 % acrylamide, stacking buffer (0.5 M tris, pH 6.8), ultra-pure water, 1 % ammonium persulfate, and 0.1 % TEMED). The protein ladder (1.5 μL) was loaded onto the gel, and electrophoresis was performed at 100 V for 1.5 h using running buffer (25 mM Tris, 0.1 % SDS, 192 mM glycine). Afterwards, the gel was washed (MeOH/ultra-pure water/acetic acid 5:4:1 *v/v/v*) for 1 h, dyed with Coomassie brilliant blue for at least 1 h, and washed again (ultra-pure water/MeOH/acetic acid 7:2:1 *v/v*/v) for 1 h. Finally, images of the gels were taken using an imager (iBright FL1500, ThermoFisher, Darmstadt, Germany).

### 2.7 Analysis of the digested proteins by LC-IMS-MS/MS

The peptide concentration of the various digests was determined using the Pierce Peptide Quantification Kit (Thermo Fisher Scientific, Bremen, Germany) following the manufacturer's instructions. For each sample, 200 ng of peptide were purified using Evotip Pure tips (Evosep Biosystems, Odense, Denmark). The tips were prepared according to the manufacturer's manual: The tips were initially rinsed with 20 μl of 100 % LC-MS grade acetonitrile (ACN) with 0.1 % LC-MS grade formic acid (FA) and centrifuged for 1 min at 800 g. Pre-conditioning of the tips was achieved by soaking them in 100 % isopropanol for 2 min. Tips were then equilibrated by adding 20 μl of 0.1 % FA, and centrifugation for 1 min at 800 g. This step was repeated. Peptides were then applied to the tips and loaded by centrifugation for 1 min at 800 g. A washing step was conducted by adding 20 μl of 0.1 % FA and centrifuging for 1 min at 800 g. For peptide storage, 200 μl of 0.1 % FA was added, followed by a final centrifugation step for 10 s at 800 g.

Peptides were separated on an Evosep One LC system using a 40 samples-per-day (40 SPD) Whisper-zoom standardized gradient (32.5 min). Eluting peptides were analysed using a timsTOF Pro ion mobility spectrometry (IMS) quadrupole time of flight mass spectrometer (Bruker Daltonics, Bremen, Germany) operated in data-dependent acquisition parallel accumulation with serial fragmentation (DDA-PASEF) mode. Ionization of peptides took place at a temperature of 180 °C and a capillary voltage of 1600 V. A blank run was performed between all samples, during which the ion mobility was automatically recalibrated. MS and MS/MS scan range was 100–1700 m/z, the ion mobility range (expressed as 1/K_0_) 0.85–1.3 V∗s/cm^2^. A polygon filtering was applied in the m/z and ion mobility area to exclude the low m/z of singly charged ions for PASEF precursor selection. Ramp and accumulation time were set to 100 ms. The number of PASEF ramps was set to 4 with a ramp rate of 9.42 Hz and a charge minimum of 0, and a maximum of 5. The quadrupole isolation width was set to 2 for m/z = 700 and 3 for m/z = 800. Collision energy was 27 eV for ion mobility (1/K_0_) 0.85 V∗s/cm^2^ and 45 eV for ion mobility (1/K_0_) 1.3 V∗s/cm^2^, respectively. Active precursor exclusion was activated with a window of 0.40 min.

FragPipe (version 23.1) (Yu et al., 2020) was used to query acquired MS/MS spectra against a *Jatropha curcas* database downloaded from the UniProt database (The Uniprot Consortium, 2025) (27684 entries). Additionally, the protein sequences of rabbit pepsin and lipase II-4, human alpha-amylase 1A, as well as trypsin, pancreatic ribonuclease, pancreatic triacylglycerol lipase, and pancreatic alpha-amylase from *Sus scrofa* were obtained from UniProt and added to the database. Decoy sequences were automatically generated by FragPipe and appended to the original database for MSFragger, as well as sequences of common contaminants. Semi-specific peptide sequence identification was performed with MS Fragger version 4.3 (Kong et al., 2017). Precursor and fragment mass tolerances were set to 20 ppm, with mass calibration and parameter optimization enabled. Protease specificity was configured for tryptic digestion, which cleaves proteins C-terminal to arginine and lysine, as well as for conditions matching pepsin, which cleaves C-terminal to phenylalanine, leucine, tyrosine, and tryptophan. Up to three missed cleavage sites were permitted, along with variable modifications including methionine oxidation and protein N-terminal acetylation, as well as fixed modifications such as carbamidomethyl cysteine. The allowed peptide length and mass ranges were 7–50 residues and 500–5000 Da, respectively. Peptide-to-spectrum matches (PSMs) were rescored by MSBooster (version 1.3.17) (Yang et al., 2023) and Percolator (version 3.7.1) (Käll et al., 2007) using retention time, ion-mobility, and MS/MS spectra predictions generated by DIA-NN (Demichev et al., 2022). Based on PSMs, identified proteins were inferred by ProteinProphet (Nesvizhskii et al., 2003). Philosopher (version 5.1.1) was used to perform FDR filtering (1 % FDR at the PSM, ion, peptide, and protein level). Quantification analysis was performed with IonQuant (version 1.11.11) (Yu et al., 2021) using default parameters. Match-between-runs (MBR) was enabled with a MBR ion FDR of 1 %. Peptides were quantified using the calculated peptide intensities given in the FragPipe output files.

### 2.8 Determination of Xuta's free amino acid absorption

Two different cell lines were selected for the transport studies: Human colon adenocarcinoma cells Caco-2 (German Collection of Microorganisms and Cell Cultures, Braunschweig, Germany) and HT29-MTX (European Collection of Authenticated Cell Cultures; Porton Down, UK). Both cell lines were cultured in Dulbecco's modified Eagle's medium (DMEM) containing (w) 4.5 g/L glucose, w 0.87 g/L stable glutamine, w 0.11 g/L sodium pyruvate, w 3.7 g/L NaHCO_3_, supplemented with 10 % (*v/v*) fetal bovine serum (FBS), 1 % penicillin/streptomycin and 1 % non-essential amino acids (100x) at 37 °C in a humidified atmosphere containing 5 % CO_2_. Medium was changed regularly every 2–3 days, and the cells were split once they reached 80–90 % confluence using trypsinization (trypsin/EDTA). Passages 15–20 were used for the cell culture studies.

To examine the transepithelial transport of amino acids and peptides, a transport experiment was conducted using a co-culture consisting of two different intestinal epithelial cell lines, Caco-2 and HT29-MTX, according to the method described by Köpsel et al. (2025). The cells were cultured at a ratio of 7:3 and seeded at a concentration of 5 × 10^4^ on Transwell® polyester membrane cell culture inserts (0.33 cm^2^ growth surface area, 0.4 μm membrane pore size, 4 × 10^6^ pores per cm^2^; Corning, USA) in 24-well plates. Apical and basolateral medium (composition as described) were added at volumes of 200 μL and 800 μL, respectively. The co-culture was differentiated for 21 days at 37 °C in a humidified atmosphere with 5 % CO_2_. The culture medium was replaced every second day until a confluent monolayer or so-called “barrier integrity” was obtained. After 21 days, the growth medium was removed, cells were washed with PBS (500 μL apical, 1000 μL basal), and incubated for 10 min at 37 °C. Subsequently, the PBS was removed. The different treatment samples were diluted with DMEM without phenol red, containing 5 μM sodium fluorescein, at a concentration of 5 mg/mL, identified as non-cytotoxic in a prior cytotoxicity assay (see supplemental material). While 200 μL of the treatment solution was applied apically to the cells, 800 μL of DMEM w/o phenol red was added to the basolateral compartment. The cells were then treated in total for 4 h at 37 °C and 5 % CO_2_. To monitor monolayer cell integrity, TEER values were measured before (0 h), after 2 h, and at the end of the experiment (4 h) (see section 2.9). Only cultures with stable values greater than 1000 Ω were considered for the transepithelial transport assessment of Xuta protein digests. Additionally, the solution in the basolateral compartment was sampled after 2 h, stored at −20 °C, and replaced with 800 μL of fresh DMEM without phenol red. After 4 h, samples from both the apical and basal compartments were collected and stored at −20 °C until further analysis.

### 2.9 Transepithelial electrical resistance (TEER) measurement

According to Srinivasan et al. (2015) the transepithelial electrical resistance (TEER) measurements were conducted to monitor the integrity of the cell layer using the Millicell ERS-2 Volt-Ohm Meter (Millipore, Bedford, MA, USA) connected to a pair of chopstick electrodes. After sterilisation and an equilibration period at room temperature, the electrode pair was placed vertically into the insert, with one electrode in the basal and one in the apical chamber. This process started with the cell-free inserts (R_blank_) and measuring the resistance across the cell layer on the semipermeable membrane (R_sample_) (Srinivasan et al., 2015). The resistance values were given in ohms (Ω), and TEER values were calculated using equation (2):

(2) $$\text{TEER}=\left(R_{\text{sample}}‐R_{\text{blank}}\right)*\text{growth}\;\text{surface}\;\text{area}$$

TEER values were measured each time before a media change. To control the monolayer cell integrity, TEER values were monitored before (0 h), after 2 h, and after the transport experiment (4 h).

### 2.10 Paracellular permeability determination

The amount of sodium fluorescein in the samples obtained from transport experiments was measured in order to evaluate transepithelial permeability through tight junctions, as described by Catanzaro et al. (2015). The sodium fluorescein concentration in the samples was evaluated using a standard calibration curve (50–2.5 μM) prepared from pure sodium fluorescein in the same medium used for the transport experiment: DMEM without phenol red (section 2.8). The absorbance of the standards as well as of the samples “basolateral after 2h”, “basolateral after 4h”, and “apical after 4h” was measured by pipetting 65 μL into a 96-well plate and reading the absorbance at 570 nm using a microplate reader. The apparent permeability coefficient (Papp) index was then calculated according to Del Mar Contreras et al. (2012) and Palumbo et al. (2008) as an expression for paracellular transport based on parameters of the transport experiment with the following equation (3):

(3) $$\text{Papp}\;\text{Index}=\frac{\Delta Q}{\Delta t}\times \frac{1}{A\times C_{0}}$$

ΔQ accumulated mass of measured sodium fluorescein in the sample (in nM)

Δt duration (in seconds)

A growth area of insert (in cm^2^)

C_0_ sodium fluorescein concentration applied in the apical compartment (in nM)

### 2.11 Determination of amino acids using HPLC-FLD

The AA content was measured in the dissolved, freeze-dried supernatants from the digested samples (section 2.4) and the basolateral compartment following the transport experiment (section 2.8). AAs were analysed using high-performance liquid chromatography with fluorescence detection (HPLC-FLD), which consisted of an autosampler AS 6.1L, a pump, a gradient former, a degasser, a column oven (all Knauer, Berlin, Germany), a fluorescence detector RF-20A (Shimadzu, Japan), and an RP column (Gemini 5 μm C18, LC Column 150 × 4.6 mm, Phenomenex, Aschaffenburg, Germany) after pre-column derivatisation with OPA (Lee and Drescher, 1978). The samples from the transport experiment were mixed with 0.1 M potassium carbonate buffer solution. Twenty microliters of this mixture were then injected into the HPLC system. The oven temperature was set to 40 °C, and gradient elution was performed using (A) 0.1 M sodium acetate buffer (pH 6.5), and (B) methanol according to the following programme: 0 min: 90 % A, 5 min: 90 % A, 40 min:40 % A, 45 min: 0 % A, 50 min: 0 % A, 55 min: 90 % A, 60 min: 90 % A. Online derivatisation was performed as follows: 20 μL of the reaction agent (100 mg OPA, 1 mL potassium borate buffer, 0.1 mL 3-meracptopropionic acid and 9 mL methanol) was added to the samples. Derivatisation was carried out for 120 s and the reaction was stopped by adding 50 μL 1 M acetic acid. AAs were detected at an excitation wavelength of 330 nm and a detection wavelength of 460 nm. A dilution series of 20–800 μM was prepared for t calibration using a 2.5 mM AA stock solution of AA containing the following amino acids: Alanine, arginine, aspartic acid, cysteine, glutamic acid, glycine, histidine, isoleucine, leucine, lysine, methionine, phenylalanine, proline, serine, threonine, tyrosine, valine, tryptophan, glutamine, and asparagine. The concentrations of the AAs were calculated for the quantification of AAs using external calibration with linear regression. The amount of AA was calculated using the molar mass (Table 1) of the individual AA and the volume of the reaction mixture and related to the sample amount using equation (4).

(4) $$m_{\text{Amino}\;\text{acid}}=\frac{c_{\text{Amino}\;\text{acid}}*V_{\text{Reaction}\;\text{volume}}*\;M_{\text{Amino}\;\text{acid}}}{m_{\text{sample}}}$$

### 2.12 Calculation of nutritionally relevant scores: DIAAS, EAA

The Digestible Indispensable Amino Acid Score (DIAAS) was calculated based on the measurable free AAs released after *in vitro* simulated digestion. The recommended AA scoring pattern for adults was adapted from the FAO report (Food and Agriculture Organization of the United Nations, 2011) (His: 15 mg/g protein, Ile: 30 mg/g protein, Leu: 59 mg/g protein, Lys: 45 mg/g protein, sulfuric AA (Met + Cys): 22 mg/g protein, aromatic AA (Phe + Tyr): 38 mg/g protein, Thr: 23 mg/g protein, Trp: 6 mg/g protein, Val: 39 mg/g protein) and was used according to the following equation (5):

(5) $$\text{DIAAS}\;\%=\frac{\text{mg}\;\text{of}\;\text{digestible}\;\text{dietary}\;\text{indispensiable}\;\text{AA}\;\text{in}\;1g\;\text{of}\;\text{test}\;\text{protein}}{\text{mg}\;\text{of}\;\text{the}\;\text{same}\;\text{digestible}\;\text{dietary}\;\text{indispensiable}\;\text{AA}\;\text{in}\;1g\;\text{of}\;\text{reference}\;\text{protein}\;}\times 100$$

The calculated DIAAS value relating to the limiting amino acids (LAAs) of each sample is presented in tabular form, alongside the LAAs and the amount of essential amino acids (EAAs) per 1 g of protein. This was calculated using equation (6):

(6) $$\text{EAA}\;\%=\frac{\Sigma \;\text{EAA}\;\left(\text{mg}\right)\;}{\Sigma \;\text{AA}\;\left(\text{mg}\right)\;}\times 100$$

### 2.13 Statistical analysis

Statistical analysis was performed using the software GraphPad Prism (version 10.2.2 for Windows; GraphPad Software, Boston, Massachusetts, USA). Data were analysed for normality of distribution by the Shapiro-Wilk test, followed by a two-way ANOVA including Tukey's multiple comparison test for normally distributed data. Unless otherwise stated, differences were considered significant when *p* < 0.05 and data are presented as the mean ± SD from at least three independent experiments.

## 3 Results and discussion

### 3.1 Protein content

**Fig. 1:**
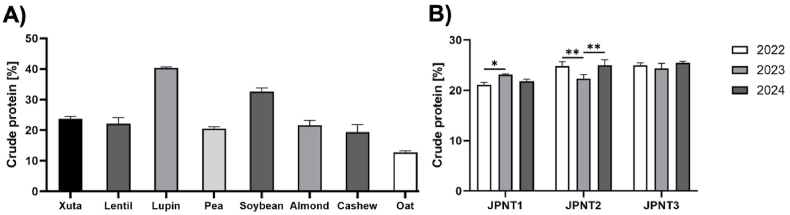
**Crude protein content (%) of different plant protein sources determined by Kjeldahl:** A) Plant protein sources (Xuta, lentil, lupin, pea, soybean, almond, cashew, oat); B) Xuta kernels of different harvest seasons 2022, 2023, and 2024 compared within each variety (JPNT1, JPNT2, and JPNT3); tested for normal distribution with Shapiro-Wilk, two-way ANOVA with Tukey's multiple comparison test, *p*∗<0.05, *p*∗∗<0.01. Bars are mean values of triplicates with standard deviation, n = 3.

Kjeldahl analysis revealed that Xuta kernels contain approximately 24 % crude protein, placing them near the median among the other alternative protein sources studied. Lupin had the highest content at 40 %, while oats had the lowest at 13 % (Fig. 1A). Among the Xuta samples, JPNT3 from 2024 harvest showed the highest protein content with 25.5 %, while JPNT1 from the 2022 harvest had the lowest at 21.1 %. Minor variations in protein content were observed both between varieties and across the 2022, 2023, and 2024 harvest years (Fig. 1B), suggesting the influence of genetic and environmental factors. Although Xuta cultivation practices remained constant, the plants were naturally exposed to varying environmental conditions, such as changes in temperature and rainfall (section 2.1), which may have affected the assimilation of nitrogen and its conversion into AAs and proteins during plant maturation (Besaliev et al., 2021; Sułek et al., 2025; Hospodarenko et al., 2018). This is supported by literature showing broad variability in Xuta protein content due to geographic and climatic differences. For example, Basha et al. (2009) reported crude protein contents ranging from 18.8 % to 34.5 % across more than 70 samples from different continents. Similar variability was also observed in Mexican Xuta samples, highlighting the combined effect of genotype and environment on protein content (Martínez-Herrera et al., 2010; Martiñón Marínez et al., 2018). Although harvest timing can affect the nutritional composition of other nuts, such as pistachios (Esmaeilpour and Shakerardekani, 2018), this factor was controlled in the present study by using samples exclusively from the July–September harvest (section 2.1), meaning it did not contribute to the observed differences in protein content. Taken together, these findings confirm the importance of environmental and agronomic factors in shaping the protein content of Xuta kernels and highlight the need to carefully consider these factors when evaluating this species as a plant-based protein source.

### 3.2 Xuta protein digestibility

#### 3.2.1 Free amino acid content

**Fig. 2:**
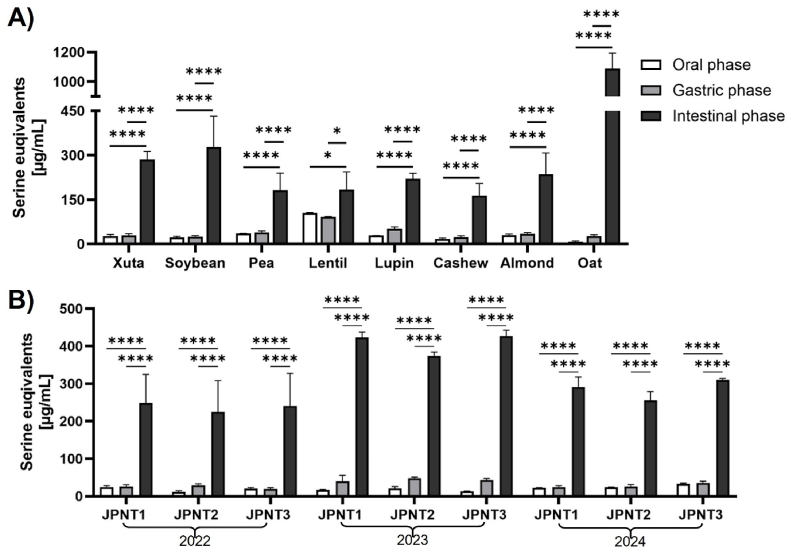
**Degree of hydrolysis expressed as serine equivalents [μg/mL] of protein sources digested according to INFOGEST - comparison of oral, gastric, and intestinal phase:** A) Plant protein sources (soybean, pea, lentil, lupin, cashew, almond, oat); B) Xuta varieties JPNT1, JPNT2, JPNT3 from different harvesting seasons (2022, 2023, 2024); Test for normal distribution with Shapiro-Wilk, two-way ANOVA with Tukey's multiple comparison test, *p*∗<0.05, *p*∗∗∗∗<0.0001. Bars represent mean values of triplicates with standard deviation (n = 3).

The degree of protein hydrolysis was assessed using the OPA method, which quantifies the number of primary amino groups in the supernatant of digested samples after they have been precipitated with perchloric acid. This method measures free amino groups, expressed as serine equivalents, to compare the protein hydrolysis across different sources. However, as OPA only detects free amino groups and excludes absorbable oligopeptides, it tends to underestimate actual protein digestibility. Therefore, it is advisable to complement OPA results with other techniques, such as gel electrophoresis or peptide analysis, to achieve a more comprehensive evaluation (Rieder et al., 2021). A significant increase in serine equivalents was observed from the oral to the gastric phase and then to the intestinal phase in all samples (Fig. 2A). The amount of intestinal serine equivalents in Xuta digests was lower than in oats and similar to that in soybeans, but higher than in the other crops. A significant increase was also evident from the oral to the gastric phase, and further to the intestinal phase, which was also significantly evident in all Xuta samples (Fig. 2B). These findings are consistent with previous studies on other plant-derived proteins, indicating that Xuta proteins undergo progressive enzymatic cleavage under simulated gastric and intestinal conditions (Rebollo-Hernanz et al., 2023; Wang et al., 2024a, 2024b). The gradual release of amino groups corresponds to pepsin activity in the gastric phase, followed by pancreatic proteases such as trypsin and chymotrypsin in the intestinal phase. The comparatively moderate release of amino groups in the oral phase reflects the absence of proteolytic enzymes, with protein breakdown limited to mechanical disruption by mastication. Here, salivary amylase targets carbohydrates (Goksen et al., 2025).

#### 3.2.2 SDS-PAGE patterns of Xuta protein hydrolysates

Protein hydrolysis was further assessed using SDS-PAGE. Samples collected after the oral (OP), gastric (GP), and intestinal (IP) digestion phases were loaded onto polyacrylamide gels and stained with Coomassie brilliant blue. A representative gel of the Xuta varieties from the 2024 harvest is shown; the reference protein gels can be found in the supplementary material (Fig. S1).

**Fig. 3:**
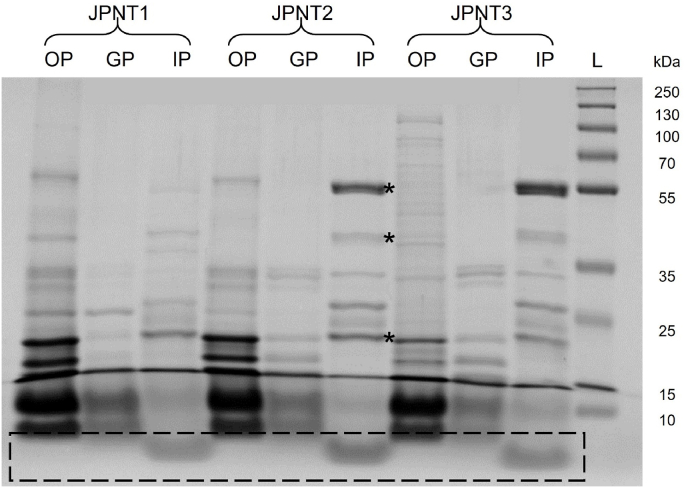
**SDS-PAGE of digested Xuta protein. Comparison of oral (OP), gastric (GP), and intestinal phase (IP):** Xuta varieties of the 2024 harvest season (JPNT1, JPNT2, JPNT3). 10 μg protein per lane. Polyacrylamide gel stained with Coomassie brilliant blue; ladder (L). ∗digestion enzymes.

A decrease in the number and intensity of protein bands within the high molecular weight range (25–250 kDa) was observed from OP to GP and then to IP, indicating the progressive enzymatic degradation of intact proteins during simulated digestion (Fig. 3). This finding is consistent with the increasing degree of hydrolysis previously quantified via the OPA assay (section 3.2.1). The degradation of high-molecular-weight proteins into low-molecular-weight proteins and peptides was evident in the labelled region (Fig. 3), which was only present in the IP samples. This suggests that the line with a molecular weight of less than 10 kDa, which could not be resolved using the applied gel composition, represents peptides. Similar protein breakdown patterns have been reported for other plant proteins during *in vitro* digestion (Sousa et al., 2020; Rebollo-Hernanz et al., 2023). Several bands remained visible in the IP samples and are marked with asterisks in Fig. 3. These correspond in size to the porcine digestive enzymes introduced during the simulated digestion process, including pancreatic amylase and lipase (∼50–54 kDa), trypsin (∼23 kDa), and pepsin (∼38 kDa). Their presence does not indicate undigested Xuta protein, but rather the presence of exogenous enzymes, as reported in other *in vitro* digestion studies (Sousa et al., 2020). The SDS-PAGE-based findings complement the chemical quantification of hydrolysis analyses and provide visual confirmation of progressive proteolysis.

#### 3.2.3 Peptide length after simulated digestion

**Fig. 4:**
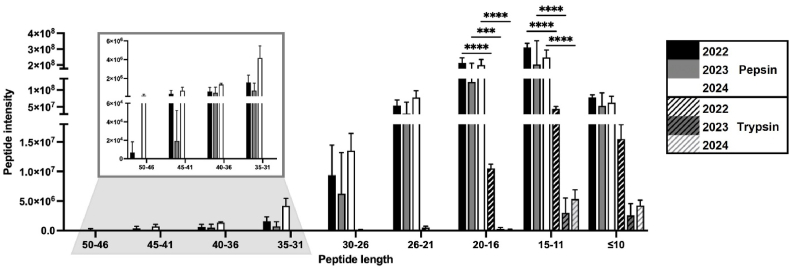
**Peptide length of digested Xuta samples from gastric phase (pepsin) and intestinal phase (trypsin) expressed as peptide intensity measured with LC-MS:** Comparison of Xuta digests after gastric phase (pepsin) with intestinal phase (trypsin) within the same harvest year (2022, 2023, 2024). Test for normal distribution with Shapiro-Wilk, two-way ANOVA with Tukey's multiple comparison test, *p*∗∗∗<0.001, *p*∗∗∗∗<0.0001. Bars represent mean values of triplicates with standard deviation (n = 3).

To characterise the peptides released during digestion, shotgun proteomics was performed on Xuta digests that had been collected after both the gastric (pepsin hydrolysis) and the intestinal phases (trypsin hydrolysis). The intensity and length of the detected peptides were analysed, with length defined by the number of amino acids (AAs). Fig. 4 shows the peptide length of the three varieties (JPNT1-3), averaged across the harvest years 2022–2024. Statistical comparisons were conducted within each harvest year between pepsin- and trypsin-mediated hydrolysis. The complete dataset is available in the supplementary material (Table S1).

Only peptides shorter than 30 AAs were detected in the trypsin-digested samples, whereas the pepsin-digested samples showed higher overall peptide intensities, including longer peptides (Fig. 4). A significant decrease in peptide length was observed after the intestinal phase compared to the gastric phase for all harvest years, particularly within the 20–16 AA and 15–11 AA length categories. These results suggest that protein degradation by trypsin is progressive and align with the increasing degree of hydrolysis shown by the OPA assay (section 3.2.1, Fig. 2B) and SDS-PAGE (section 3.2.2, Fig. 3). Similar digestion patterns have been reported for milk proteins (Atallah et al., 2020). Peptides comprising fewer than 10 AAs are generally considered bioavailable, as they can be directly absorbed or further hydrolysed by brush border enzymes (Rieder et al., 2021). Furthermore, most bioactive peptides are short, typically comprising 2–20 AAs (Chakrabarti et al., 2018). Therefore, Xuta protein hydrolysates, which are rich in short peptides, could represent a valuable source of bioactive peptides, warranting further exploration. The reduction in peptide length and intensity observed during simulated *in vitro* digestion thus suggests enhanced digestibility of Xuta proteins (Fig. 4). To further resolve the distribution of peptide sizes at different digestion stages, complementary methods such as size-exclusion chromatography could be employed (Zimmermann et al., 2025).

### 3.3 Free amino acid release after digestion

**Fig. 5:**
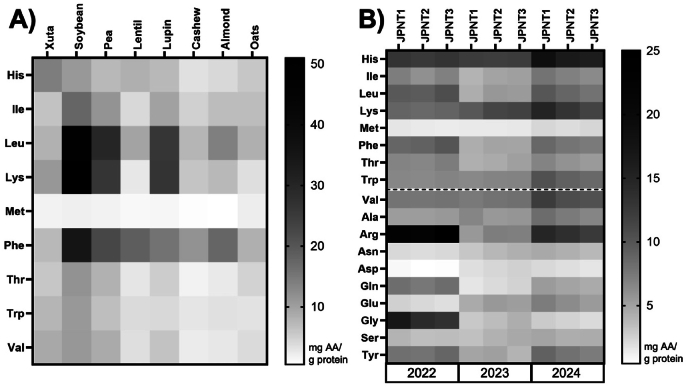
**Amino acids (mg AA/g sample protein) of protein sources digested according to INFOGEST detected by HPLC-FLD:** A) Essential AA of Xuta, soybean, pea, lentil, lupin, cashew, almond, oat; B) AA of Xuta varieties (JPNT1, JPNT2, JPNT3) from harvesting seasons 2022, 2023, 2024.

The AAs present in the supernatant of the digested samples were identified and quantified using HPLC-FLD. The results are expressed as the concentration of each AA in milligrams per gram of protein in the corresponding raw material subjected to digestion (Fig. 5).

Across all plant-based protein sources, methionine was the least abundant EAA, except for oats, where lysine showed the lowest concentration (Fig. 5A). This observation aligns with the known composition of storage proteins: legumes such as soybeans, peas, and lupins predominantly contain globulins, which are typically rich in lysine and leucine but deficient in methionine. Nuts contain globulins to a lesser extent, resulting in comparatively lower lysine levels, while cereals like oats are mainly composed of glutelins with small amounts of globulins. This explains their moderate lysine content (Singh et al., 2025; Azizi et al., 2025; Klose et al., 2009).

In the 2022, 2023, and 2024 JPNT1–3 Xuta samples, methionine consistently remained the limiting EAA, while histidine and lysine were comparatively abundant. In line with earlier studies on native Xuta kernels (Martínez-Herrera et al., 2006; Makkar and Becker, 2009), arginine dominated the total AA profile (Fig. 5B). Interestingly, glutamic acid, which is often described as the most abundant AA in native Xuta, did not rank highest after digestion. This pattern can be explained by the enzyme-specific cleavage preferences during digestion, as the most important gastrointestinal proteases (pepsin, trypsin, chymotrypsin) primarily cleave aromatic or basic residues rather than acidic residues such as glutamate, thereby reducing the chance of complete release (Fu et al., 2021). Notably, tryptophan, often excluded from AA profiles due to analytical challenges, was successfully quantified in all digested Xuta samples. Given its physiological role as a serotonin precursor and its status as an indispensable dietary AA, this is a relevant finding (Richard et al., 2009). When comparing the AA profiles of digested Xuta kernels to values reported in the literature, it is important to note that previous studies often investigated different varieties, cultivation regions, and harvest years, which limits direct comparability. Both genetic and environmental factors are known to influence AA composition. For example, Senger et al. (2017) demonstrated a pronounced effect of growing region on the Xuta kernel profiles.

In this study, the difference between harvest years was more pronounced than the differences between varieties. Serine concentrations remained relatively stable across all samples (range: 3.07–4.53 mg/g protein), while arginine levels were significantly higher in the 2022 and 2024 harvests than in the 2023 harvest (Fig. 5B). Similar trends were observed for leucine, phenylalanine, and tyrosine, which supports the hypothesis that variation in the climate between growing seasons affects protein metabolism and nitrogen allocation. Arginine, in particular, affects as a nitrogen storage compound in plants and accumulates in response to abiotic stress such as drought, which may explain the observed interannual variability (Winter et al., 2015). This interpretation is supported by heatmap analysis and aligns with findings in other crops, where environmental factors (e.g., temperature, rainfall, and fertilization) have been shown to impact AA composition (Besaliev et al., 2021; Sułek et al., 2025; Hospodarenko et al., 2018). While genotype remains important, studies on grains emphasise the significant influence of agrotechnical and climatic conditions (Jaśkiewicz and Szczepanek, 2018; Zhang et al., 2016). Although no detailed meteorological data were available for the Xuta cultivation regions in this study (section 2.1), the observed annual differences emphasise the importance of considering the environmental context when evaluating the nutritional quality of novel plant protein sources. In addition, differences in peptide release patterns observed among harvest years and varieties (see supplemental material, Table S1) may have further contributed to the variability in the post-digestive AA profiles, as peptide composition and length can influence the extent of AA liberation and absorption.

### 3.4 Xuta AA profile after transport experiment

**Fig. 6:**
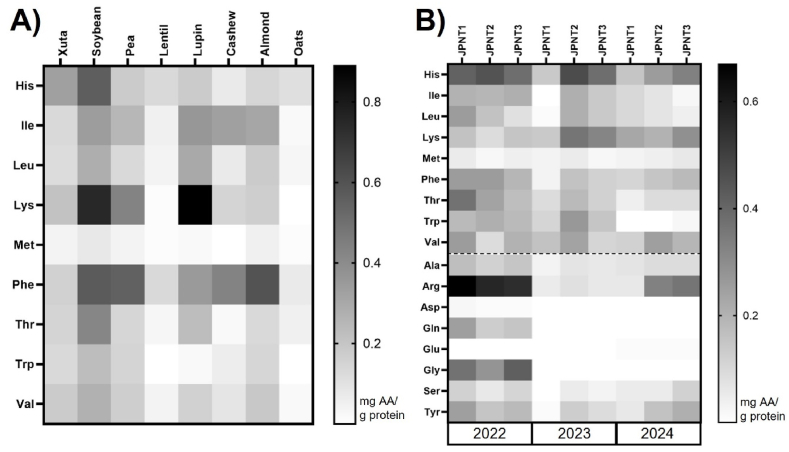
**Amino acids (mg AA/g sample protein) of protein sources digested according to INFOGEST, followed by transport experiment using Caco2/HT29-MTX cells detected by HPLC-FLD:** A) Essential AA of Xuta, soybean, pea, lentil, lupin, cashew, almond, oat; B) AA of Xuta varieties (JPNT1, JPNT2, JPNT3) from harvesting season 2022, 2023, 2024. A transport experiment was performed by treating the co-culture with 5 mg/mL freeze-dried supernatant of the digested alternative protein source.

The transport of released free amino acids (AAs) from digested Xuta samples was assessed using an *in vitro* transport experiment, building on the peptide length and post-digestion AA profiles. A co-culture of Caco-2 and HT29-MTX cells was used to simulate the human intestinal epithelium using a non-cytotoxic concentration of digested samples (Figure S2). After the incubation period, the AA concentrations in the basolateral compartment, representing the absorbed fraction, were quantified (Fig. 6). This analysis complements the digestion data by estimating the minimum bioavailable fraction, represented by the free AAs released that successfully crossed the epithelial layer. AAs that could be released by brush-border peptidases *in vivo*, were not considered due to the inherent limitations of the *in vitro* model. The limiting AA (LAA) remained unchanged after the transport experiment compared to the post-digestion profile (Fig. 5). Methionine consistently showed the lowest abundance across all samples, except in oats, where lysine exhibited the lowest abundance (Fig. 6A). Across Xuta varieties and harvest years, methionine was generally the least transported EAA, with exceptions in JPNT1 (2023) and JPNT3 (2024), where isoleucine had the lowest basolateral concentration (Fig. 6B). Isoleucine, a branched-chain EAA, is absorbed apically via B^0^AT1 and exported basolaterally through large AA transporters such as LAT2 and LAT4 (Bröer, 2023). However, these transporters are not specific to isoleucine, but also transport leucine, valine, phenylalanine, and methionine. Such overlapping substrate specificity can cause competition, which could explain the lower isoleucine transport in some samples (Bröer and Gauthier-Coles, 2022).

Histidine was generally the most abundant EAA post-transport, except in JPNT1 (2024), where lysine predominated (Fig. 6B). As a positively charged EAA, histidine is absorbed via b^0,+^AT, which exchanges cationic for neutral AAs. These neutral AAs can then be reabsorbed by B^0^AT1, indicating a sequential rather than competitive mechanism (Bröer and Gauthier-Coles, 2022). On the basolateral side, histidine transport via y^+^LAT1 also depends on neutral AA exchange, which may contribute to the high histidine levels observed (Bröer, 2023).

Similar to the digestion results (Fig. 5), AA transport profiles revealed greater variation between harvest years than between varieties, suggesting that environmental conditions may have a more substantial influence on AA bioavailability than genetic factors. However, varietal differences were evident in 2024, specifically for JPNT1 and JPNT3, where the limiting EAA was different (lysine versus isoleucine). This suggests that while environmental factors dominate, genotype can still influence competitive transport dynamics. Overall, these findings highlight the importance of considering both genetic and environmental influences when evaluating the nutritional quality of plant proteins, particularly with regard to post-digestive bioavailability.

Tryptophan in JPNT1 (2024) and JPNT2 (2024), glycine and aspartic acid in all varieties from 2023 to 2024, serine for JPNT1 (2023), and glutamic acid from 2022 were below the limit of quantification and are therefore colored white.

### 3.5 Effect of Xuta protein digests on intestinal epithelium integrity

#### 3.5.1 Transepithelial electrical resistance (TEER)

**Fig. 7:**
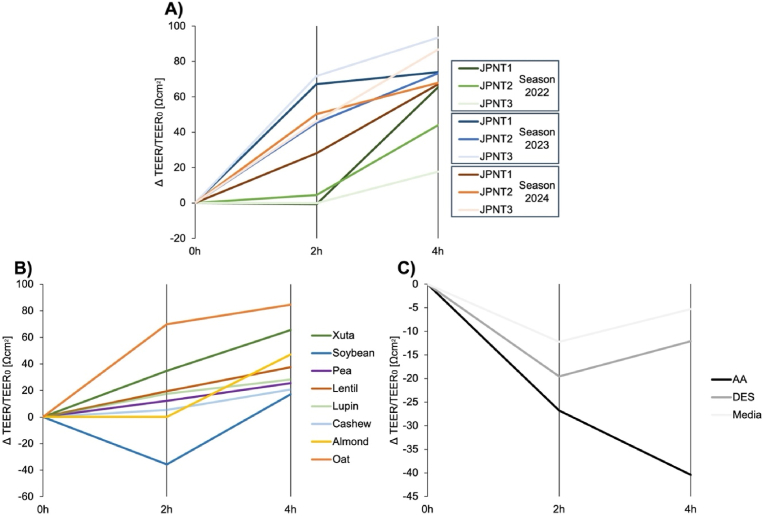
**Change (Δ) of TEER values in relation to the start TEER value at 0 h (Ωcm^2^) measured during the transport experiment with co-culture of Caco-2 and HT29-MTX cells; after 2 h and 4 h:** A) Xuta varieties (JPNT1, JPNT2, JPNT3) from different harvesting seasons 2022, 2023, 2024. B) Plant protein sources: Xuta, soybean, pea, lentil, lupin, cashew, almond, and oat. C) Controls: Media control, digestive electrolyte solution (DES) containing all electrolyte solutions used during INFOGEST procedure, and amino acid standard (AA) obtained from a commercial supplier. Samples were used with the non-cytotoxic concentration of 5 mg/mL. Values are means of triplicate using different cell passages without standard deviation, n = 3.

The TEER experiment was performed using non-cytotoxic concentrations (see supplemental material) to evaluate the effect of digested protein samples on the integrity of the intestinal barrier. Measurements were taken at 0, 2, and 4 h, and expressed relative to the baseline value (Fig. 7).

Digested Xuta proteins from 2023 to 2024 induced a steady increase in TEER throughout the 4 h, indicating an enhanced intestinal epithelial cell integrity (Fig. 7A). The 2022 Xuta sample exhibited a comparable response, albeit with a delayed onset after 2 h. Increased TEER values suggest reduced membrane permeability, which could be due to enhanced tight junction assembly or remodeling, improved cell-to-cell adhesion, altered ionic conditions, or reduced cellular stress (Shaughnessey et al., 2022; Gonschior et al., 2022; Bonnans et al., 2014).

TEER increases were comparable for most other plant-based protein digests, except for almond and soybean (Fig. 7B). In cells treated with almonds, TEER remained stable for the first 2 h before increasing, suggesting a delayed effect. By contrast, soybean treatment initially decreased TEER, indicating temporary weakness possibly due to oxidative or inflammatory stress, tight junction loosening, cellular stress, or apoptosis due to toxic substances, mechanical stress, or fluid shear force (Shaughnessey et al., 2022; Gonschior et al., 2022; Bonnans et al., 2014). However, TEER values recovered within 4 h, indicating an adaptive response (Fig. 7).

For both control treatments, digestive electrolyte solution (DES) and pure media, TEER initially decreased before increasing again. Notably, the free AA control showed a continuous decrease in TEER over the 4 h period (Fig. 7C), suggesting that free AA alone cannot enhance barrier integrity. Instead, the digestion-derived peptides or accompanying matrix components generated during gastrointestinal digestion appear to mediate protective or barrier-strengthening effects. Overall, these results highlight that digested plant proteins, including Xuta, can positively influence intestinal barrier function. This adds a physiological dimension to their nutritional value, as they provide bioactive components beyond free AAs.

#### 3.5.2 Paracellular permeability

Sodium fluorescein is a low-molecular-weight compound that primarily crosses the intestinal epithelium via the paracellular route, making it a well-established marker for tight junction dynamics (Köpsel et al., 2025). In this experiment, sodium fluorescein was added to the apical side of the cell monolayer during treatment, with basolateral samples were collected after 2 and 4 h. The Papp index was calculated to indicate transport activity (section 2.10).

**Fig. 8:**
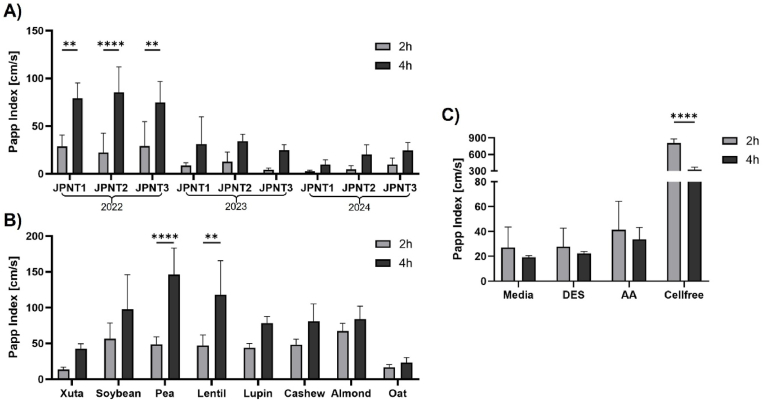
**Transport of sodium fluorescein in co-culture transport experiment (Caco-2 & HT29-MTX cells) expressed as Papp Index:** A) Xuta varieties (JPNT1, JPNT2, JPNT3) from different harvesting season (2022, 2023, 2024); B) Plant protein sources (soybean, pea, lentil, lupin, cashew, almond, oat); C) Controls: Media control, digestive electrolyte solution (DES) containing all electrolyte solutions used during INFOGEST procedure, free AA control, cell-free wells. All were tested with a concentration of 5 mg/mL. Sharpiro-Wilk test followed by Two-way ANOVA with Tuckey's multiple comparison test, *p*∗∗∗∗<0.0001. Values as average of triplicate with standard deviation n = 3.

For cells treated with digested Xuta and other plant-based protein alternatives, the Papp index increased from 2 h to 4 h, although not all changes were statistically significant (Fig. 8). This increase suggests a temporary modulation of the tight junctions, allowing enhanced paracellular passage of small molecules such as sodium fluorescein. However, this does not necessarily indicate weakening of the barrier, especially since TEER values increased in these samples over the same period (Fig. 7), and may instead reflect a controlled adjustment of tight junction dynamics.

Importantly, digested proteins, including free AAs and small peptides, are predominantly absorbed via the transcellular route, using specific transporters such as members of the SLC family (for AAs) and PEPT1 (for di- and tripeptides) (Bröer, 2023). Therefore, the physiological relevance of a moderately increased Papp index should be interpreted with caution, since it does not directly reflect the efficiency of nutrient uptake, but rather provides information on general barrier permeability.

In contrast, all control samples (media, DES, the free AA control, and the cell-free blank) showed a decrease in Papp index from 2 h to 4 h, indicating that the paracellular pathway tightened over time. For the media control and DES, this trend was supported by a simultaneous increase in TEER (Fig. 7C). Notably, the AA control was the only sample for which TEER values decreased continuously throughout the 4 h treatment period (section 3.5.1, Fig. 7C).

The combined TEER and fluorescein permeability data suggest that the continuous decline in TEER observed for the free AA control does not reflect a structural disruption of tight junctions. Since TEER primarily reflects the ionic conductance across the monolayer, it can change rapidly due to alterations in ion channel or transporter activity or osmotic effects, whereas fluorescein permeability measures the paracellular diffusion of small hydrophilic solutes (Srinivasan et al., 2015; Shaughnessey et al., 2022; Gonschior et al., 2022; Bonnans et al., 2014; Köpsel et al., 2025). These two mechanisms can therefore be regulated independently and do not necessarily correlate, as tight junction proteins can differentially control ion and solute passage depending on their expression and organization within the polarized epithelium (Karakocak et al., 2023; Zeller et al., 2015).

In contrast, the results obtained for the Xuta digests and those of other plant-based protein sources indicate that digestion-derived matrix components modulate barrier properties in a more complex and physiologically active manner. Specifically, these components may stimulate cellular signaling pathways that lead to a reorganization of tight junction proteins (Karakocak et al., 2023; Zeller et al., 2015). This selective modulation suggests that digested protein fractions, especially those from Xuta, promote a physiologically adaptive and protective epithelial state rather than compromising barrier integrity.

### 3.6 Nutritional scores

To evaluate the quality of the protein after digestion, the digestible indispensable amino acid score (DIAAS) and the amount of EAAs per gram of protein were calculated for the Xuta varieties (Table 2) based on the sum of free AAs released after digestion. This approach reflects the fraction of AAs immediately available for absorption but excludes short peptides that would *in vivo* be hydrolysed by brush-border enzymes (Rieder et al., 2021). Consequently, the presented values represent a minimum digestibility and may underestimate the total absorbable AAs. The average values for Xuta were then compared to those of selected plant-based protein alternatives (Table 3).

**Table 2:** **Limiting amino acids (LAA), the digestible indispensable amino acid score (DIAAS) [%], the amount of essential amino acids (EAA) per 1 g protein [%]:** Comparison of the Xuta varieties (JPNT1, JPNT2, JPNT3) from harvesting season 2022, 2023, 2024, including the range of values (min-max).

	2022			2023			2024			Xuta
	JPNT1	JPNT2	JPNT3	JPNT1	JPNT2	JPNT3	JPNT1	JPNT2	JPNT3	
LAA	Methionine			Methionine			Methionine			Met
**DIAAS (%)**	6.66	5.92	5.35	5.61	5.64	6.07	9.48	8.26	9.26	5.35–9.48
**EAA (%)**	49.8	50.9	51.2	61.4	60.4	60.3	60.9	60.4	61.1	49.8–61.1

**Table 3:** **Limiting amino acid (LAA), the digestible indispensable amino acid score (DIAAS) [%] and the amount of essential amino acids (EAA) per 1g protein [%]:** Comparison of Xuta, soybean, pea, lentil, lupin, cashew, almond, oat.

	Xuta	Soybean	Pea	Lentil	Lupin	Cashew	Almond	Oat
LAA	Methionine							Lysine
**DIAAS (%)**	6.92	7.28	6.46	3.81	4.96	2.17	2.09	7.12
**EAA (%)**	57.0	49.7	50.7	31.4	51.8	36.5	36.1	42.6

Methionine was identified as the LAA in all Xuta samples, as well as in most of the reference proteins, except for digested oat, where lysine was limiting (Table 3). This finding is consistent with the post-digestive AA profile, in which methionine and lysine were the least abundant AA in the respective samples (Fig. 5). The highest DIAAS values among the Xuta varieties were obtained for the 2024 harvest (Table 2), which highlights the impact of agronomic and climatic conditions on protein quality and AA availability. On average, Xuta (6.92 %) was comparable to soybean (7.28 %), pea (6.46 %), and oat (7.12 %), and higher than lentil (3.81 %), lupin (4.96 %). Cashew and almond exhibited the lowest values in the reference group (Table 3).

These absolute DIAAS values are lower than the values reported in the literature for plant proteins, e.g., soybean (85–91 %), lupin (68 %), pea and oat (57 %), and lentil (53 %) (Goksen et al., 2025; Han et al., 2020; Herreman et al., 2020; Nosworthy et al., 2018). This discrepancy results primarily from the calculation approach used here, in which only the free AAs released after digestion were considered as the accessible fraction present in absorbable di- and tripeptides, as well as those that could be released by brush-border peptidases *in vivo*, were not included due to the inherent limitations of the *in vitro* method compared with *in vivo* digestion (Rieder et al., 2021). This tends to underestimate the total digestible AA pool and therefore resulted in lower DIAAS values compared to fully hydrolysed reference data. Furthermore, cysteine data were not available, so DIAAS was calculated using methionine alone, while the FAO reference patterns (Food and Agriculture Organization of the United Nations, 2011) use the combined sulphur AAs (methionine and cysteine). This applies to all tested proteins equally, making the relative comparisons valid. The consistent identification of methionine as the LAA underlines its importance in plant protein formulation and suggests that combining Xuta with methionine-rich sources such as hemp, brown rice, corn, or potatoes would improve its AA profile (Gorissen et al., 2018).

In terms of total EAA content, the lowest value was observed in the 2022 Xuta harvest, while the average across all Xuta samples was the highest among the tested plant-based proteins (Table 3). This highlights Xuta's potential as a high-quality alternative protein source, provided the raw material, particularly the protein content, remains consistent (Fig. 1B). For industrial applications such as protein concentrates or isolates, standardised quality control and analytical specifications will be essential.

## 4 Conclusion

This study investigated how varieties and harvest years influence the protein digestibility, epithelial transport properties, and amino acid (AA) profiles of Xuta kernels compared to other plant-based protein sources. The findings revealed considerable variation in crude protein content and AA profiles across Xuta harvests, suggesting that environmental factors, such as climatic conditions, are arguably more important than genotype. *In vitro* digestion of Xuta proteins showed progressive enzymatic hydrolysis, with shotgun proteomics indicating a significant reduction in peptide length and intensity during the intestinal phase compared to the gastric phase. This indicates effective trypsin-mediated digestion of the Xuta protein, suggesting high bioaccessibility of Xuta AAs, which was also consistent with the free AAs released, and reflected in the SDS-PAGE patterns.

Epithelial transport studies showed that digested Xuta proteins enhanced transepithelial electrical resistance (TEER), indicating strengthening of the epithelial barrier, while concurrently inducing a moderate increase in paracellular permeability without compromising the integrity of the epithelium. In contrast, the control sample, a mixture of free AAs, did not improve barrier function, underlining the physiological relevance of digestion-derived peptide fractions from Xuta.

Quantitative AA analysis after digestion and epithelial transport showed that methionine was the most consistently limiting essential AA, while arginine was among the most abundant. Further transport experiments showed the selective translocation of AAs, with evidence of transporter-specific preferences and potential competition. Further, Xuta demonstrated a high average content of essential amino acids and favourable digestibility. These findings underline Xuta's nutritional potential as a sustainable source of plant protein and emphasise the significant impact of environmental conditions on its protein quality and AA composition. The digestion and epithelial transport results provide valuable insights into the bioavailability and physiological relevance of Xuta protein. To address the consistent limitation of methionine, combining Xuta with methionine-rich sources is recommended to create balanced, plant-based protein formulations. Furthermore, standardised quality controls are essential to ensure consistent raw material quality for food industry applications. Finally, investigating short peptide sequences from Xuta protein hydrolysates and the screening of their potential biological activities could further demonstrate the value of this promising, eco-friendly crop and provide solutions to certain health issues.

## Author contributions: CRediT

Mona Grünwald: Writing – review and editing, Writing – original draft, Visualization, Methodology, Investigation, Formal analysis.

Nabil Adrar: Writing – review and editing, Supervision.

George Francis: Writing – review and editing, Resources.

Matthias: Writing – review and editing, Investigation.

Nils Rugen: Writing – review and editing, Investigation.

Hans-Peter Braun: Writing – review and editing, Investigation.

Tuba Esatbeyoglu: Writing – review and editing, Supervision, Resources, Conceptualization, Funding acquisition, Investigation, Project administration.

## Funding sources

The publication of this article was funded by the Open Access Fund of Leibniz Universität Hannover.

## Declaration of competing interest

The authors declare that they have no known competing financial interests or personal relationships that could have appeared to influence the work reported in this paper.

## Data Availability

The mass spectrometry proteomics data as well the complete FragPipe analysis output have been deposited to the ProteomeXchange Consortium (Deutsch et al., 2023) via the Mass Spectrometry Interactive Virtual Environment (MassIVE) repository with the MassiVE dataset identifier MSV000099049 (doi: 10.25345/C56W96N93).
