# Supplementary material for: A comparative study of the nutritional and physiological potential of Xuta (edible Jatropha curcas L.) protein: Insights into its digestibility and effects on the intestinal barrier
Source: Curr Res Food Sci. 2025 Dec 1;12:101257. doi: 10.1016/j.crfs.2025.101257 (PMC12753251; doi:10.1016/j.crfs.2025.101257)
Supplement: Multimedia component 1 [file mmc1.docx]

**A Comparative Study of the Nutritional and Physiological Potential of Xuta (Edible *Jatropha curcas* L.) Protein: Insights into Its Digestibility and Effects on the Intestinal Barrier**

Mona Grünwald (0009-0007-6767-4113)^1^; Nabil Adrar (0000-0003-3723-3003)^1^; George Francis^2^, Nils Rugen (0000-0002-9297-4560)^3^, Matthias Döring (0009-0006-7357-9350)^3^, Hans-Peter Braun (0000-0002-4459-9727)^3^, Tuba Esatbeyoglu (0000-0003-2413-6925)^1^

^1^ Department of Molecular Food Chemistry and Food Development, Institute of Food and One Health, Gottfried Wilhelm Leibniz University Hannover, Am Kleinen Felde 30, 30167 Hannover, Germany; gruenwald@foh.uni-hannover.de; adrar@foh.uni-hannover.de; esatbeyoglu@foh.uni-hannover.de

^2^ Jatropower AG, Haldenstrasse 5, CH-6340, Baar, Switzerland

^3^ Department of Plant Proteomics, Institute of Plant Genetics, Gottfried Wilhelm Leibniz University Hannover, Herrenhäuser Straße 2, 30419 Hannover, Germany

Corresponding author: Prof. Dr. Tuba Esatbeyoglu, email: esatbeyoglu@foh.uni-hannover.de, Tel.: +49511762 5589

**Supplemental material**

SDS-PAGE

Samples collected after oral (OP), gastric (GP), and intestinal (IP) digestion phases were loaded side by side on acrylamide gels and stained with Coomassie brilliant blue.


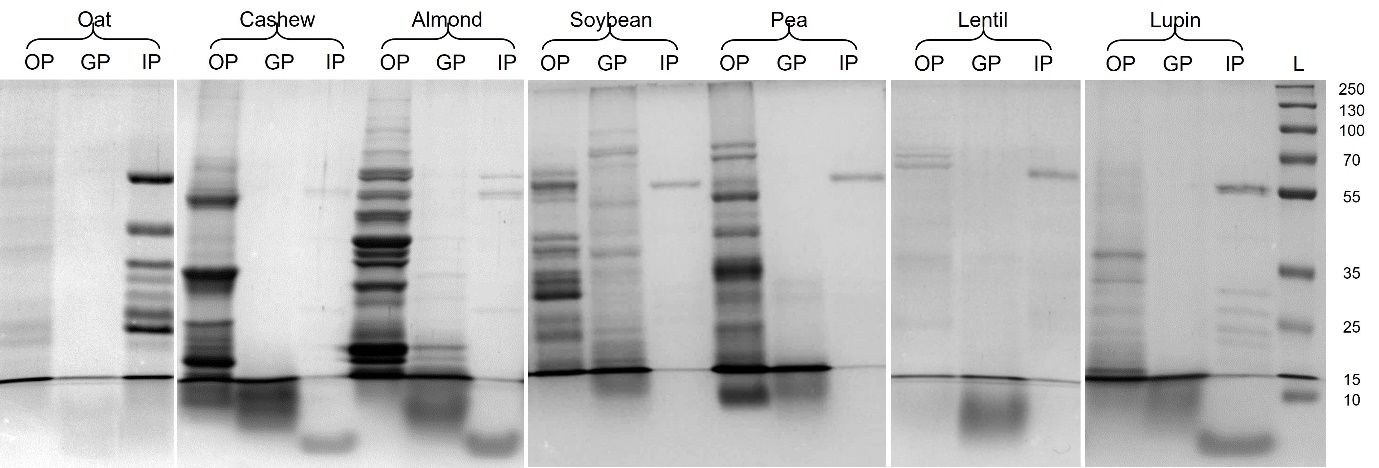


**Figure S1: SDS-PAGE of reference samples digested according to INFOGEST - comparison of oral (OP), gastric (GP), and intestinal phase (IP):** Plant protein sources (oat, cashew, almond, soya, pea, lentil, lupin). 10 µg protein per lane. Polyacrylamide gel stained with Coomassie brilliant blue; ladder (L).

Peptide length

The peptides released during digestion were analysed using shotgun proteomics. The measurements were performed on Xuta digests that had been collected after both the gastric (pepsin hydrolysis) and the intestinal phases (trypsin hydrolysis). The intensity and length of the detected peptides were analysed, with length defined by the number of amino acids (AAs).

**Table S1: Peptide length of digested Xuta samples from gastric phase (pepsin) and intestinal phase (trypsin) expressed as peptide intensity measured with LC-MS:** Comparison of Xuta digests after gastric phase (pepsin) with intestinal phase (trypsin) for the harvest years (2022, 2023, 2024).

|  | **Pepsin / Gastric phase** | | | **Trypsin / Intestinal phase** | | |
| --- | --- | --- | --- | --- | --- | --- |
| **Peptide length** | **JPNT1** | **JPNT2** | **JPNT3** | **JPNT1** | **JPNT2** | **JPNT3** |
|  | **2022** | | | | | |
| 50-46 | 20077 | 0 | 0 | 0 | 0 | 0 |
| 45-41 | 778897 | 86897 | 267113 | 0 | 0 | 0 |
| 40-36 | 381730 | 1114645 | 305998 | 0 | 0 | 0 |
| 35-31 | 2207827 | 1789522 | 722499 | 0 | 0 | 0 |
| 30-26 | 14796467 | 8645353 | 4671822 | 70629 | 209935 | 0 |
| 26-21 | 70487468 | 53230432 | 38114484 | 696989 | 583127 | 213051 |
| 20-16 | 240454564 | 220527476 | 177697020 | 11354186 | 10085812 | 10129111 |
| 15-11 | 326422623 | 324778539 | 278752641 | 45101881 | 35803467 | 50923233 |
| ≤10 | 77058366 | 86029939 | 71154647 | 18471435 | 12773504 | 17299273 |
|  | **2023** | | | | | |
| 50-46 | 0 | 0 | 0 | 0 | 0 | 0 |
| 45-41 | 57342 | 0 | 0 | 0 | 0 | 0 |
| 40-36 | 197833 | 1152059 | 97988 | 0 | 0 | 0 |
| 35-31 | 1612452 | 129381 | 379095 | 0 | 0 | 0 |
| 30-26 | 14225967 | 3017601 | 1503851 | 7743 | 0 | 0 |
| 26-21 | 69080398 | 12214968 | 10771243 | 29198 | 22644 | 10250 |
| 20-16 | 206379546 | 30431169 | 132699416 | 521505 | 50366 | 309670 |
| 15-11 | 317434565 | 35326331 | 259762824 | 2902433 | 534269 | 5548823 |
| ≤10 | 57276398 | 12671035 | 89163264 | 1805569 | 1086804 | 4822424 |
|  | **2024** | | | | | |
| 50-46 | 9999 | 152728 | 335132 | 0 | 0 | 0 |
| 45-41 | 1114321 | 471422 | 485304 | 0 | 0 | 0 |
| 40-36 | 1198415 | 1414796 | 1447874 | 0 | 0 | 0 |
| 35-31 | 4033145 | 5518033 | 2970840 | 0 | 0 | 0 |
| 30-26 | 28424605 | 14020698 | 16194373 | 0 | 0 | 0 |
| 26-21 | 100680736 | 65002880 | 68203477 | 23617 | 0 | 23754 |
| 20-16 | 240816182 | 182477281 | 176865024 | 103720 | 24944 | 236288 |
| 15-11 | 291215705 | 253158237 | 198902957 | 4453149 | 4428714 | 7135491 |
| ≤10 | 76505828 | 70230808 | 40646725 | 4241276 | 3316714 | 5149750 |

Cytotoxicity

The cytotoxicity of samples used in the transport experiment was determined by MTT assay using different concentrations, while in Figure S2 the non-cytotoxic concentration is presented with 5 mg/mL.


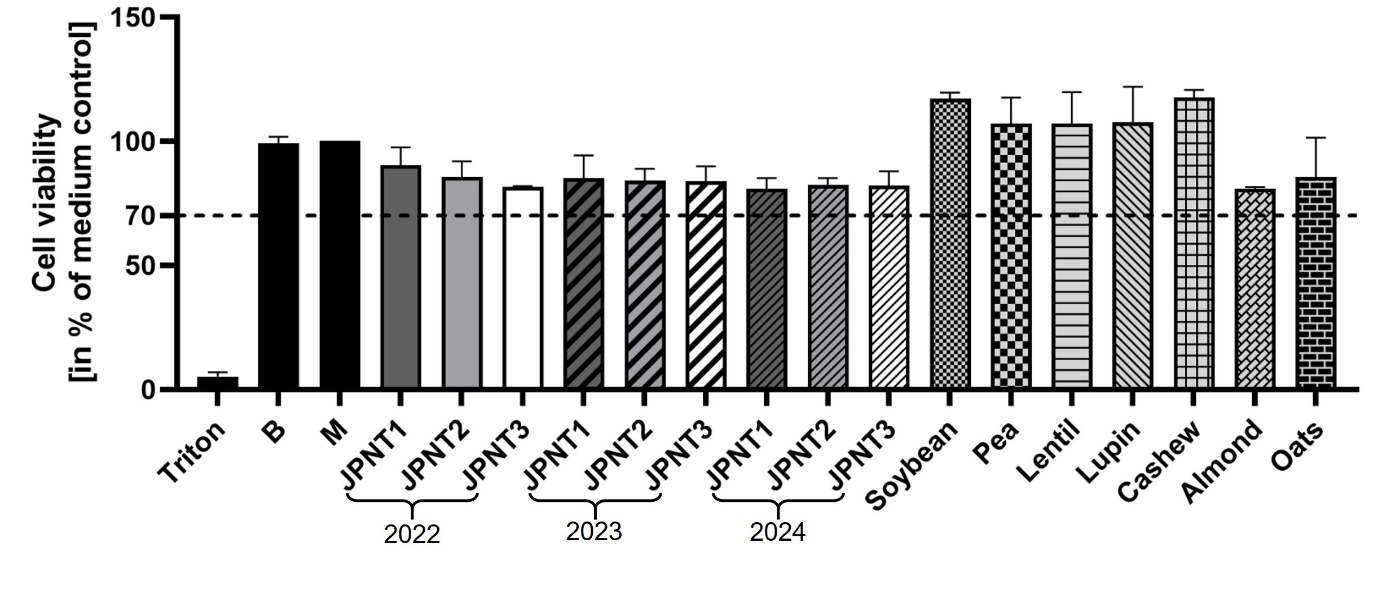


**Figure S2: Non-cytotoxic concentration (5 mg/mL) determined by MTT assay of digested freeze-dried supernatants from different alternative protein sources:** Xuta varieties (JPNT1, JPNT2, JPNT3) from different harvesting seasons (2022, 2023, 2024), soybean, pea, lentil, lupin, cashew, almond, oats. MTT Assay was performed by using the co-culture model of Caco-2 and HT29-MTX cells, including a positive control with Triton^TM^ X, as well as solvent control (B) and medium control (M). Values are presented as mean with standard deviation, n=3.
